# Effects of Intensive Vibratory Treatment with a Robotic System on the Recovery of Sensation and Function in Patients with Subacute and Chronic Stroke: A Non-Randomized Clinical Trial

**DOI:** 10.3390/jcm11133572

**Published:** 2022-06-21

**Authors:** Mª Pilar Rodríguez-Pérez, Patricia Sánchez-Herrera-Baeza, Roberto Cano-de-la-Cuerda, Lucía Rocío Camacho-Montaño, Sergio Serrada-Tejeda, Marta Pérez-de-Heredia-Torres

**Affiliations:** Department of Physical Therapy, Occupational Therapy, Rehabilitation and Physical Medicine, King Juan Carlos University, Avenida de Atenas s/n, CP, Alcorcon, 28922 Madrid, Spain; pilar.rodriguez@urjc.es (M.P.R.-P.); roberto.cano@urjc.es (R.C.-d.-l.-C.); lucia.camacho@urjc.es (L.R.C.-M.); sergio.tejeda@urjc.es (S.S.-T.); marta.perezdeheredia@urjc.es (M.P.-d.-H.-T.)

**Keywords:** focal vibration, functionality, hand, rehabilitation, robotic, stroke, upper limb, vibration

## Abstract

Background: Sensory–motor deficits are frequent and affect the functionality after stroke. The use of robotic systems to improve functionality and motor performance is advisable; therefore, the aim of the present study was to evaluate the effects of intensive, high-frequency vibration treatment administered with a robotic system in subacute and chronic stroke patients in terms of upper limb sensitivity, motor function, quantity and quality of movement, and quality of life. Methods: A simple-blind, non-randomized controlled trial was conducted. The control group received conventional rehabilitation treatment and the experimental group received robotic treatment with an Amadeo^®^ robot in addition to their conventional rehabilitation sessions. Results: Intragroup analysis identified significant improvements in the experimental group in hand (*p* = 0.012), arm (*p* = 0.018), and shoulder (*p* = 0.027) sensitivity, as well as in motor function (FMA-UEmotor function, *p* = 0.028), integration of the affected limb (MAL-14amount scale, *p* = 0.011; MAL-14How well scale, *p* = 0.008), and perceived quality of life (SIS-16, *p* = 0.008). The measures between the control and experimental groups showed statistically significant differences in motor performance and spontaneous use of the affected limb (MAL-14amount scale, *p* = 0.021; MAL-14How well scale, *p* = 0.037). Conclusions: Intensive, high-frequency vibration with a robotic system, in combination with conventional intervention, improves the recovery of upper limb function in terms of quantity and quality of movement in patients with subacute and chronic stroke.

## 1. Introduction

Stroke is one of the leading causes of death and disability in the world [1]. The sequelae can be multiple and variable, but among the most frequent deficits are motor, somatosensory, and perceptual disorders in relation to the upper limb. As a result, the ability to perform the activities of daily living (ADLs) may be severely compromised in stroke patients [2]. Between 55 and 85% of subjects have paresis in the upper limb, causing motor limitation and significant difficulties in incorporating that limb in ADLs [3,4,5], as well as a decrease in health-related quality of life [6].

Several studies have highlighted the strong influence and relationship between motor and sensory deficits as the most frequent symptoms after stroke [5,6]. Approximately 50% of stroke patients experience sensory impairment, especially tactile and proprioceptive discrimination [7]. Patients with sensory deficits, in addition to the motor component, are nine to thirteen times more at risk of functional impairment compared to those without, regardless of other associated clinical deficits [7,8].

The planning of rehabilitation programmes for these patients involves a complex multidisciplinary approach to motor, sensory, and/or neuropsychological deficits that requires early and effective treatment that has not yet been resolved [9]. According to the rates of deficits recorded in Western countries, at six months after stroke, more than 60% will have a hand that is not functional in performing ADLs [10]. In this sense, new technologies would allow the incorporation of elements, such as intensity and repetition of functional tasks, that are considered key in stroke rehabilitation. Among them, robotics [11] and virtual reality [12] are positioned as widely accepted complementary tools in stroke rehabilitation [13].

The Amadeo^®^ manual robotic system (Tyromotion GmbH, Graz, Austria, 2016) is a so-called end-effector robot that includes a proprioceptive treatment function as it uses sensors placed on the fingertips that provide a vibratory stimulus of different frequencies; this could be interesting for the sensory treatment of this type of patient [14]. Previous studies, such as a preliminary one by Stain et al. [15] and a more recent one by Sale et al. [16], have explored the use of this system in the treatment of motor and functional deficits in people after stroke [1,15]. However, to our knowledge, the effect of the robotic-therapy-mediated sensory programme on the recovery of upper limb sensation, motor function, quantity and quality of movement, and health-related quality of life has not been previously studied using this technology in stroke patients.

The main objective of the present study was to determine the effects of a conventional rehabilitation programme, in combination with the Amadeo^®^ manual robotic system, on the affected upper limb in patients with subacute and chronic stroke compared to a control group not receiving robotic therapy.

## 2. Materials and Methods

### 2.1. Design

This was a single-blind, non-randomised clinical trial, following the Transparent Reporting of Evaluations with Non-Randomized Designs (TREND). The study was approved by our University Ethics Committee (505202012620). All the participants provided written informed consent before study participation.

### 2.2. Participants

Patients with stroke were recruited from Hospital Los Madroños and Hospital Universitario 12 de Octubre (Madrid, Spain).

The inclusion criteria were: age between 30 and 80 years, hemiparesis or hemiplegia of the left upper limb, right-handedness, alterations in sensation in the affected upper limb as a consequence of the lesion determined in the neurological assessment, being in the subacute stage (between three and six months after the event) or chronic stage of the disease (more than six months after the stroke) [17], and acceptance and signature of the informed consent form by the patient.

Exclusion criteria were: aphasia, apraxia, no limitation of mobility and/or sensibility in the affected upper limb, concomitant diseases affecting mobility and/or sensibility, cognitive impairment with scores below 24 points on the Mini-Mental State Examination (MMSE) [18], and a disease course of less than three months.

Non-probabilistic sampling of consecutive cases was used. The patients were distributed according to their geographical allocation into a control group who received conventional rehabilitation treatment and an experimental group who received robotic treatment with Amadeo^®^ in addition to their conventional rehabilitation.

Data collection was conducted between September 2020 and August 2021. Once the patient had agreed to the study and filled in the informed consent form, the assessment was carried out. Data were collected through a customised and anonymised booklet on the patient’s medical history with socio-demographic data, neurological examination results, and the scores of the different scales administered. The administration of the tests was carried out by an external evaluator trained in the use of these measures, blinded to the intervention received by the subjects, and they were administered in the same order: Semmes–WeinsteinMonofilaments^®^, Fugl–Meyer Assessment Upper Extremity Scale, Motor Activity Log, and Stroke Impact Scale. Participants were scheduled in the same time slot, outside their usual treatment.

After the initial assessment, the intervention was carried out in the relevant neurological rehabilitation units of each hospital.

### 2.3. Intervention

The experimental group received vibration robotic therapy with the Amadeo system^®^ version 5.1. This end-effector robotic system features a support arm and straps that are placed on the forearm and wrist with the patient in a seated position; on each of the fingers, plasters with a small, magnetised plate are placed on the fingertips where they are anchored to the levers of the system. The programme provides a vibratory proprioceptive stimulus of different frequencies and intensity [15]. The control group underwent conventional neurological rehabilitation (CNR) sessions three times a week for one and a half months (24 sessions). The treatment of the experimental group began, following the published stimulation recommendations [19,20], with the vibration session with the Amadeo^®^ robotic system version 5.1 with a vibration duration of 20 min at the maximum frequency (60 Hz). The intensity was determined according to the tolerance of the subject. Following the robotic therapy, the patient received his/her usual occupational therapy treatment, with a duration of 45 min (Vibration + CNR).

The control group received 24 CNR sessions three times a week with a duration of 45 min. Conventional neurological rehabilitation in the control group followed the same protocol as in the experimental group, based on the principles of neurorehabilitation treatment for upper limbs as determined in the clinical practice guideline management of stroke rehabilitation [21], and based on the practice of repetitive and specific tasks, with a duration of between two and twenty weeks and regular ADL training.

### 2.4. Measures

The outcome measures used in this study pre- and post-treatment were: Semmes–Weinstein Monofilaments^®^, Fugl–Meyer Assessment Upper Extremity Scale, Motor Activity Log, and Stroke Impact Scale.

The Semmes–Weinstein Monofilaments^®^ is the most common and accurate instrument for sensory assessment in the stroke population [22]. The Touch-Test hand kit (North Coast Medical, Inc., Morgan Hill, CA, USA, 2011) was used, consisting of five monofilaments. These nylon fibres are identified with numbers within a range of 1.65 to 6.65 assigned by their manufacturers, with the thickest being interpreted as the worst result [23]. The investigator explained the test procedure, and, then, with the patient’s eyes closed, slowly placed each filament at a perpendicular angle against the skin until it arched, held it in place for 1.5 s, and then slowly removed it. The procedure was started with the 2.83 filament, which is defined as the limit for normal sensation; if the 2.83 filament was not felt, thicker filaments were applied in the same way; a value of 7 was assigned when no monofilament was felt by the patient. The fibres were applied to all areas corresponding to the dermatomes of the upper limb on the anterior and posterior side [24,25,26].

The Fugl–Meyer Assessment Upper Extremity Scale is one of the most commonly used and specific tests to assess motor and sensory impairment in stroke patients [27]. The assessment includes measurements of voluntary movement, speed, coordination, and reflex activity, using an ordinal scale applied to each item. This scale has a specific section for the assessment of upper limb sensation [28]. The maximum score is 66/100 for the upper limb. The assessment includes the measurement of voluntary movement, speed, coordination, and reflex activity through an ordinal scale applied to each item: 0: cannot be performed, 1: partially, and 2: completely. The Fugl–Meyer Assessment Upper Extremity score is classified into severe motor impairment with less than 32 points, moderate between 32 and 47, or mild with 48 points or more. This scale also has a specific section for the assessment of upper limb sensation, with a maximum score of 12 points, integrating surface sensation and proprioception; the score is 0 when absent, 1 when decreased, and 2 when normal [29,30].

Motor Activity Log assesses the functionality and motor performance of the affected upper limb specifically, as well as its spontaneous use and integration in quantity and quality in the performance of ADLs [31]. This measure is based on patient report and not a direct observational assessment of motor performance. Scoring is completed on a 6-point ordinal scale (range 0–5), on which half-scores can also be given, with higher scores indicating better performance: maximum score is 5 for each item, and the scores for each subscale of quality and quantity of movement are added together to provide a maximum score of 70 for each subscale [32]. The psychometric properties of Motor Activity Log indicate reliability coefficients of 0.96 for the quantity and quality scales in its Spanish version and adequate psychometric properties.

The Stroke Impact Scale is a scale specifically for patients with brain damage and measures the impact it has had on their quality of life. It is a valid and reliable measure for a diverse group of patients following a stroke. Each item is rated using a 5-point Likert scale. The patient rates their difficulty in completing each item, where: 1 = inability to complete the item and 5 = no difficulty experienced at all [33]. The Stroke Impact Scale-16 version has been further validated and can be administered to the patient through a retrospective interview over the past two weeks about their difficulty in performing ADLs [34].

### 2.5. Statistical Analysis

Statistical analysis was performed with SPSS 27.0 for Windows (Copyright© 2022 IBM Corp. Released 2020. IBM SPSS Statistics for Windows. Armonk, NY, USA: IBM Corp). For qualitative variables, the number of cases present in each category and the corresponding percentage were calculated, and, for quantitative variables, the mean and standard deviation were calculated. Normality was tested with the Shapiro–Wilk test. Differences were considered statistically significant at 95% confidence level (*p* < 0.05). To study the differences in scores from pre- to post-treatment in both groups, Wilcoxon tests were performed. For the mean difference analysis of the pre to post scores between the two groups, the Mann–Whitney U statistic was calculated. Subsequently, differences between groups were estimated by calculating the effect size and using Rosenthal’s r-statistic (r). Interpretation was based on Cohen’s (1988) guidelines for interpreting the results (0.2 = small effect, 0.5 = medium effect, and 0.8 or higher = large effect) [35]. To correct for type I error, multivariable regression models were created to identify the influence of confounding independent variables on post-treatment scores that were significant in the comparative analysis.

## 3. Results

**Figure 1:**
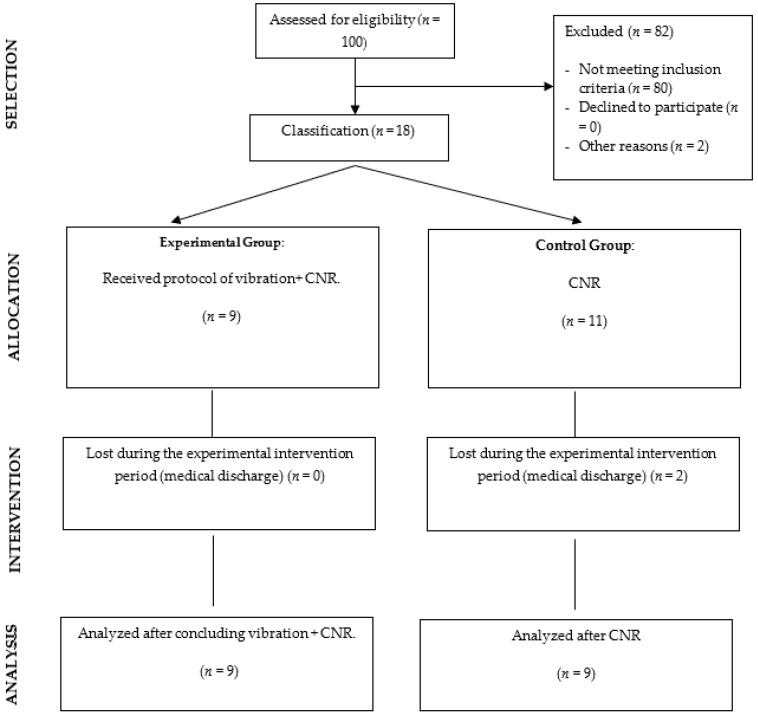
Flow diagram.

The final sample consisted of 18 participants; nine subjects received vibration + CNR and nine received CNR. There was a loss of two subjects, who were discharged before the completion of treatment (Figure 1). The age range of the study population was 45–85 years, 67% were male, and the mean number of years since disease diagnosis was 8.83 months, with a range of 3–14 months since diagnosis (Table 1).

Table 2 shows the analysis of intra-group differences before and after treatment of the variables related to the sensory and pain assessment of the study. In the control group, no statistically significant differences were identified. However, the patients in the experimental group showed significant results after the intervention process in the sensitivity of the hand, arm, and shoulder (*p* < 0.05). In both groups, no significant post-treatment differences were identified in the variables related to upper limb joint tenderness and pain measured by FMA-UE.

In the rest of the dermatomes of the upper extremity analysed in the experimental group, statistically significant differences (*p* < 0.05) were found in all of them, except in the posterior distal area of the forearm (*p* = 0.203). In the control group, post-intervention evaluation showed a worsening in the rest of the hand dermatomes, except in the dorsal medial area (Supplementary Table S1).

Multivariate regression analyses identified that, in the variables that showed statistically significant differences in the experimental group, the variables age, sex, and duration of the disease did not influence the post-treatment results. However, the baseline level of pre-treatment sensation had a positive influence (*p* < 0.05) (Supplementary Tables S2–S4).

Table 3 shows the analysis of intra-group differences before and after treatment for variables related to motor and functional assessment. In the control group, no statistically significant differences were evident. However, in the experimental group, the results showed significant differences related to motor function, quantity and quality of movement on the MAL-14 scale, as well as perceived quality of life (*p* < 0.05). All these differences showed a medium effect size (>0.50). The rest of the variables analysed showed no significant differences between the control and experimental groups.

Multivariate regression analyses identified that, in the variables that showed statistically significant differences in the experimental group, the variables age, sex, and duration of the disease did not influence the post-treatment results. However, the baseline level of motor function, quality of life, and motor performance level before treatment had a positive influence (*p* < 0.05) on the post-treatment outcome (Supplementary Tables S5–S8).

Table 4 shows the analysis of intergroup differences between the control and experimental groups. In the post-intervention analysis, the results showed statistically significant differences in the quantity (*p* = 0.021) and quality (*p* = 0.037) on the MAL-14 scale of the experimental group compared to the control group (*p* < 0.05) after treatment. Both differences showed a median effect size (>0.50). For the rest of the variables analysed, no differences were identified between the control and experimental groups.

Multivariate regression analyses identified that, in the variables that showed statistically significant differences in the experimental group, the variables age, sex, and duration of the disease did not influence the post-treatment results. The baseline level of motor performance level before treatment confirms a positive influence (*p* < 0.05) on the post-treatment outcome (Supplementary Tables S9 and S10).

## 4. Discussion

To our knowledge, there are currently no previously published studies on the effects of high frequency vibration with the Amadeo^®^ power version 5.1 device in terms of sensitivity, motor function, quantity and quality of movement of the affected upper limb in their ADLs, and health-related quality of life in subacute and chronic stroke patients.

The intra-group results of this study show that the combined vibration + CNR treatment led to significant post-treatment improvements in hand, forearm, and shoulder sensitivity with respect to the group that received only CNR. Additionally, in the post-intervention analysis, the results evidenced statistically significant differences in quantity (*p* = 0.021) and quality (*p* = 0.037) on the Motor Activity Log scale in favour of the experimental group compared to the control group. These clinical findings are in agreement with the previous literature on the mechanisms of action of sensory stimulation and cortical representation [34], and in terms of neuroplasticity and motor learning [36,37], as the robotic system could provide a combination of haptic and proprioceptive interaction to the motor outputs [20,38]. This pilot study suggests that the treatment of sensory function could contribute to enhance the effects of conventional treatment in this type of patients [28].

Although the results were significantly better in the intervention group, our data did not show significant differences in sensitivity between the control and experimental groups. Other authors, such as Sang et al. [39], who intervened in the chronic phase of stroke, through thirty minutes of focal vibratory stimulation (120 Hz) on the forearm musculature for six sessions three times a week, found no significant differences in the assessment of sensitivity, in agreement with our results and using the same assessment instrument. However, these authors performed their intervention for two weeks. Wei et al. [40] showed that the effects of vibration in healthy subjects produced changes in the cortex and were better when intensive treatment was carried out for 10 days or more, and more long-term changes in cortical excitability were observed. In this sense, our results could suggest that the improvements may be due to the intensity of the treatment proposed to stroke patients. In this sense, different authors, also focusing on high focal frequency vibration with other types of non-robotic portable devices and similar protocols, have described positive effects on motor aspects [41,42].

The available evidence on the treatment of the upper limb after stroke using exoskeleton-based robots and end-effectors, including the Amadeo^®^ robotic device, has focused on the recovery of motor function, while the effects on the quality and quantity of use and integration of upper limb movement in ADLs have not been previously analysed [16,43,44,45]. Calabro et al. [43] applied 25 min of motor training therapy with the Amadeo^®^ robot for 40 sessions (five days a week) but included patients in the chronic phase and only found benefits in terms of motor function, as measured by Fugl–Meyer Assessment upper extremity. The intervention of the study by Sale et al. [16] was based on the application of a total of 40 min of motor training assisted by the same robot for 20 sessions (four days a week) in acute phase patients, and their described results were only in terms of motor function.

In agreement with these authors, our results found intragroup benefits in terms of motor function in the experimental group. However, no significant differences were found between groups. In this sense, our results observed intragroup differences, as well as differences between the control and experimental groups, in the quality and quantity of upper limb movement and spontaneous use in ADL, so this could demonstrate that, by introducing vibratory stimulation as a complementary tool to conventional neurological rehabilitation, more significant improvements in the functional integration of the affected upper limb are produced. Our results coincide with studies such as that by Cosimo et al. [46] in which they applied focal muscle vibration at 300 Hz for 12 sessions (three times a week) to patients with stroke in the chronic phase. Their results were positive in motor function and, although they administered functional measures different from this study, such as the functional independence measure (FIM) and the QuickDASH tool, in line with our results, they found improvements in upper limb function. However, the quantity and quality of movement of the affected upper limb in ADLs was not assessed.

When performing the regression analysis, no statistically significant differences were observed in the comparative analyses both within and between groups in the variables age, sex, and duration of the disease, but they were obtained for the baseline state, indicating that patients with better baseline levels have higher post-treatment scores.

In addition to the benefits of the system on sensory–motor components, in this trial, intra-group improvements are observed in favour of the experimental group with respect to health-related quality of life, as measured by the SIS-16 scale. These results could be in line with the study by Scalhal et al. [46] with chronic hemiparetic patients after stroke, in which correlations were found between sensory and motor functions of the upper limb with the ability to perform functional activities. We can, therefore, hypothesise that there could be a correlation between the motor domains measured by the Motor Activity Log scale and the patient’s subjective perception of efficiency in their ADLs, as well as with the ability to spontaneously use their affected arm [47]. In other words, the better the quantity and quality of use of their arm, the better the perception of efficiency in their ADLs and, therefore, in the perception of quality of life. Our results, although they cannot prove this hypothesis, provide insight into how motor and sensory aspects influence patient functionality. Future studies could focus in depth on how these aspects are associated due to the clinical importance of sensory training in the functional integration of the upper limb in ADLs and its relation to perceived quality of life [47].

Our study suffers from a number of limitations. Firstly, we highlight the small sample size, the impossibility of randomising the sample, as well as the absence of previous studies that provide information on the ideal robotic vibration protocol. In addition, there was heterogeneity in the sample with respect to the degree of clinical involvement. Future studies should be conducted taking into account the clinical baseline of the sample for a better understanding of the real effects of the experimental protocol in stroke patients compared to a conventional treatment. On the other hand, we cannot conclude that the positive effects achieved are exclusively due to the application of robotic therapy or to the cumulative effect of working time in the experimental group. Finally, it was not possible to perform a follow-up discharge assessment of the participants included in the study. However, despite these limitations, the present study has allowed a first analysis of the possible benefits of vibration with a robotic system in conjunction with conventional neurological rehabilitation in patients who have suffered subacute and chronic stroke in a hospital setting.

## 5. Conclusions

The results of this study indicate that high frequency vibratory stimulation in combination with conventional neurological rehabilitation led to intragroup improvements in terms of hand, forearm, and shoulder sensation, motor function, the quantity and quality of movement, as well as perceived quality of life in stroke patients. Intergroup differences showed that the combination of robotic therapy and CNR produced differences in terms of the quantity and quality of movement during the performance of their ADLs in subacute and chronic stroke compared to conventional rehabilitation. Future work should corroborate these findings by including larger sample sizes and follow-up assessments.

## Figures and Tables

**Table 1:** Socio-demographic characteristics of the sample.

	CNR	Vibration + CNR	Total
	*n* = 9	*n* = 9	*n* = 18
Age M (SD)	72.89 (10.203)	66.56 (9.876)	69.72 (10.272)
Range of age (years)	54–85	45–77	45–85
Sex [N (%)]			
- *Male*	4 (44%)	8 (89%)	12 (67%)
- *Female*	5 (56%)	1 (11%)	6 (33%)
Disease duration (months) M (SD)	8.44 (4.639)	9.222 (3.382)	8.83 (3.959)
Range of disease duration (months)	3–14	4–12	3–14

*Note:* M: mean; SD: standard deviation; CNR: conventional neurological rehabilitation.

**Table 2:** Sensitive and pain assessment pre- and post-treatment in control and intervention group. Intragroup comparison.

Variable			Control Group			Experimental Group		
			Median (IQR)	*p*-Value	Rosenthal’s r	Median (IQR)	*p*-Value	Rosenthal’s r
SEMMES–WEINSTEIN MONOFILAMENT TEST	Hand	Pre	6.65 (3.39)	0.593	0.12	5.57 (4.19)	0.012 *	0.60
		Post	5.98 (3.39)			4.47 (4.07)		
	Forearm	Pre	5.65 (3.39)	1.000	0.00	4.56 (3.39)	0.161	0.33
		Post	6.15 (3.39)			4.56 (13.75)		
	Arm	Pre	6.65 (3.39)	0.276	0.25	4.65 (3.39)	0.018 *	0.56
		Post	6.65 (3.39)			4.43 (4.19)		
	Shoulder	Pre	6.65 (2.69)	0.180	0.31	4.56 (3.39)	0.027 *	0.53
		Post	6.65 (3.39)			4.31 (4.19)		
FMA-EU	Sensation	Pre	4.00 (10.00)	0.180	0.31	6.00 (6.00)	0.172	0.32
		Post	6.00 (10.00)			6.00 (10.00)		
	Joint pain	Pre	20.00 (22.00)	0.221	0.28	20.00 (7.00)	0.106	0.38
		Post	20.00 (12.00)			20.00 (7.00)		

*Note:* FMA-UE, Fugl–Meyer assessment of the upper extremity. Data are expressed as median and interquartile range (IQR). * *p*-value < 0.05 using the Wilcoxon test for related samples.

**Table 3:** Motor and functional assessment pre- and post-treatment in control and experimental group. Intragroup comparison.

Variable			Control Group			Experimental Group		
			Median (IQR)	*p*-Value	Rosenthal’s r	Median (IQR)	*p*-Value	Rosenthal’s r
**FMA-EU**	Motor function	Pre	33.00 (57.00)	0.249	0.27	14.00 (54.00)	0.028 *	0.52
		Post	33.00 (55.00)			18.00 (56.00)		
	Passive joint motion	Pre	20.00 (22.00)	0.167	0.32	20.00 (19.00)	0.461	0.17
		Post	21.00 (22.00)			19.00 (11.00)		
**MAL-14**	Amount scale	Pre	0.00 (54.00)	0.144	0.34	15.00 (38.00)	0.011 *	0.60
		Post	2.00 (62.00)			21.00 (51.00)		
	How well scale	Pre	0.00 (55.00)	0.109	0.37	14.00 (29.00)	0.008 *	0.63
		Post	2.00 (62.00)			18.00 (46.00)		
**SIS-16**	Pre		53.00 (49.00)	1.000	0.00	46.00 (42.00)	0.008 *	0.62
	Post		53.00 (49.00)			55.00 (46.00)		

*Note*: MAL-14, Motor Activity Log-14; FMA-UE, Fugl–Meyer Assessment upper extremity; SIS-16, Stroke Impact Scale-16. * *p* value < 0.05 using the Wilcoxon test for related samples.

**Table 4:** Comparison of outcome scores between the experimental group and the control group.

Variable			Median (Interquartile Range)		*p*-Value	Rosenthal’ r
			Control	Experimental		
			Group	Group		
Post	Semmes–Weinstein Monofilament Test	Hand	5.98 (3.39)	4.47 (4.07)	0.076	0.41
		Forearm	6.15 (3.39)	4.56 (13.75)	0.231	0.28
		Arm	6.65 (3.39)	4.43 (4.19)	0.060	0.44
		Shoulder	6.65 (3.39)	4.31 (4.19)	0.077	0.43
	FMA-EU	Sensation	6.00 (10.00)	6.00 (10.00)	0.449	0.17
		Joint Pain	20.00 (12.00)	20.00 (7.00)	0.655	0.10
		Motor Function	33.00 (55.00)	18.00 (56.00)	0.894	0.03
		Passive joint motion	21.00 (22.00)	19.00 (11.00)	0.304	0.24
	MAL-14	Amount scale	2.00 (62.00)	18.00 (46.00)	0.021 *	0.55
		How well scale	2.00 (62.00)	18.00 (46.00)	0.037 *	0.50
	SIS-16		53.00 (49.00)	55.00 (46.00)	0.546	0.14

*Note:* FMA-UE, Fugl–Meyer Assessment upper extremity; MAL-14, Motor Activity Log-14; SIS-16: Stroke Impact Scale-16. * *p*-value < 0.05 using Mann–Whitney test for unrelated samples.

## Supplementary Materials

The following supporting information can be downloaded at: https://www.mdpi.com/article/10.3390/jcm11133572/s1, Table S1. Sensibility assessment pre and post treatment in control and experimental group. Table S2. Multivariable regression model of the SW Monofilaments_Hand_ post-treatment score in the experimental group. Table S3. Multivariable regression model of the SW Monofilaments_Arm_ post-treatment score in the experimental group. Table S4. Multivariable regression model of the SW Monofilaments_Shoulder_ post-treatment score in the experimental group. Table S5. Multivariable regression model of the FMA-UE_Motor Function_ post-treatment score in the experimental group. Table S6. Multivariable regression model of the MAL-14_Amount scale_ post-treatment score in the experimental group. Table S7. Multivariable regression model of the MAL-14_How well scale_ post-treatment score in the experimental group. Table S8. Multivariable regression model of the SIS-16 post-treatment score in the experimental group. Table S9. Multivariable regression model of the MAL-14_Amount scale_ post-treatment score between groups. Table S10. Multivariable regression model of the MAL-14_How well scale_ post-treatment score between groups.
