# Supplementary material for: Effects of Intensive Vibratory Treatment with a Robotic System on the Recovery of Sensation and Function in Patients with Subacute and Chronic Stroke: A Non-Randomized Clinical Trial
Source: J Clin Med. 2022 Jun 21;11(13):3572. doi: 10.3390/jcm11133572 (PMC9267489; doi:10.3390/jcm11133572)
Supplement: Supplementary file 1 [file jcm-11-03572-s001.zip › jcm-1773868-supplementary.pdf]

Effects of Intensive Vibratory Treatment with a Robotic System on the Recovery of Sensation and Function in Patients with Subacute and Chronic Stroke: A Non-Randomized Clinical Trial

Mª Pilar Rodríguez-Pérez, Patricia Sánchez-Herrera-BaezaRoberto Cano-de-la-Cuerda, Lucía Rocío Camacho-Montaño, Sergio Serrada-Tejeda and Marta Pérez-de-Heredia-Torres

Department of Physical Therapy, Occupational Therapy, Rehabilitation and Physical Medicine, King Juan Carlos University, Avenida de Atenas s/n, CP, 28922 Alcorcon, Madrid, Spain

Supplementary material

1. Supplementary Table S1. Sensibility assessment pre and post treatment in control and experimental group.
2. Supplementary Tables S2–S8. Multivariate regression models of significant variables in post-treatment scores in the experimental group.
3. Supplementary Tables S9 and S10. Multivariable regression models of the dependent variables in the post-treatment scores between groups.

Supplementary Table S1. Sensibility assessment pre and post treatment in control and experimental group.

			**Control n=9**			**Intervention n=9**		
			**Median (IQR)**	**Z**	**p value**	**Median (IQR)**	**Z**	**p value**
**SEMMES-WEINSTEIN MONOFILAMENT TEST**								
**HAND**	**Ulnar Palmar**							
		Pre	6.65 (3.39)	−0.447	0.655	6.65 (4.19)	−2.375	**0.018**
		Post	6.65 (3.39)			4.31 (3.84)		
	**Radial Palmar**							
		Pre	6.65 (3.39)	−0.447	0.655	4.65 (4.19	−2.032	**0.042**
		Post	6.65 (3.39)			4.31 (4.19		
	**Medial Palmar**							
		Pre	6.65 (3.39)	−0.447	0.655	4.65 (4.19)	−2.032	**0.042**
		Post	6.65 (3.39)			4.56 (4.19)		
	**Ulnar Dorsal**							
		Pre	6.65 (3.39)	−0.447	0.655	6.56 (4.19)	−2.207	**0.027**
						4.31 (4.19)		

		Post	6.65 (3.39)					
	**Radial Dorsal**							
		Pre	6.65 (3.39)	−1.069	0.285	6.56 (4.19)	−2.527	**0.012**
		Post	4.65 (3.39)			4.31 (3.84)		
	**Medial Dorsal**							
		Pre	6.65 (3.39)	−0.447	0.655	4.56 (4.19)	−2.371	**0.018**
		Post	4.65 (3.39)			4.31 (4.19)		
**FOREARM**	**Proximal anterior**							
		Pre	6.65 (3.39)	0.000	1.000	4.56 (3.39)	−2.375	**0.018**
		Post	6.65 (3.39)			4.31 (4.19)		
	**Distal anterior**							
		Pre	6.65 (3.39)	0.000	1.000	4.65 (3.39)	−2.524	**0.012**
		Post	6.65 (3.39)			4.31 (3.84)		
	**Distal posterior**							
		Pre	6.65 (3.39)	0.000	1.000	4.65 (3.39)	−1.272	0.203
		Post	6.65 (3.39)			4.56 (4.19)		
	**Proximal posterior**							
		Pre	4.65 (3.39)	0.000	1.000	4.65 (3.39)	−2.371	**0.018**
		Post	4.65 (3.39)			4.31 (4.19)		
**ARM**	**Anterior**							
		Pre	6.65 (3.39)	−1.000	0.317	4.65 (3.39)	−2.371	**0.018**
		Post	6.65 (3.39)			4.31 (3.39)		
	**Posterior**							
		Pre	6.65 (3.39)	−1.414	0.157	4.65 (3.39)	−2.207	**0.027**
		Post	6.65 (3.39)			4.56 (4.19)		
**SHOULDER**	**Anterior**							
		Pre	6.65 (3.39)	−1.000	0.317	4.56 (3.39)	−2.207	**0.027**
		Post	6.65 (3.39)			4.31 (4.19)		
	**Posterior**							
		Pre	7.00 (2.69)	−1.342	0.180	4.56 (3.39)	−2.207	**0.027**
		Post	6.65 (3.39)			4.31 (4.19)		

Note: Data are expressed as median and interquartile range (IQR). *P value < 0.05 using the Wilcoxon test for related samples.

Supplementary Table S2. Multivariable regression model of the SW $Monofilaments_{Hand}$ post-treatment score in the experimental group.

	**SW $Monofilaments_{Hand}$** (*post-treatment*)		
	**β (SE)**	***t***	***p value***
**Sex (Male vs Female)**	0.51 (0.71)	0.72	0.512
**Age (years)**	−0.09 (0.02)	−1.14	0.314
**Disease duration**	−0.14 (0.07)	−1.84	0.139
**SW $Monofilaments_{Hand}$** *(pre-treatment)*	0.61 (0.14)	4.22	**0.013**
**R^2 (%)**	88.1%		
**Model**	F (4.17)= 15.82, **$p < 0.05$**		

Note: SW: Semmes-Weinstein; β: regression coefficient; SE: standard error.

Supplementary Table S3. Multivariable regression model of the SW $Monofilaments_{Arm}$ post-treatment score in the experimental group

	**SW $Monofilaments_{Arm}$** (*post-treatment*)		
	**β (SE)**	***t***	***p value***
**Sex (Male vs Female)**	−0.43 (1.04)	−0.41	0.699
**Age (years)**	0.04 (0.04)	1.10	0.331
**Disease duration**	0.0 (0.12)	0.82	0.456
**SW $Monofilaments_{Arm}$** *(pre-treatment)*	1.09 (0.24)	4.48	**0.011**
**R^2 (%)**	70.7%		
**Model**	F (4.17)= 5.831, $p > 0.05$		

Note: SW: Semmes-Weinstein; β: regression coefficient; SE: standard error.

Supplementary Table S4. Multivariable regression model of the SW $Monofilaments_{Shoulder}$ post-treatment score in the experimental group.

	**SW $Monofilaments_{Shoulder}$** (*post-treatment*)		
	**β (SE)**	***t***	***p value***
**Sex (Male vs Female)**	0.26 (0.84)	0.31	0.772
**Age (years)**	−0.02 (0.03)	−0.81	0.460
**Disease duration**	−0.04 (0.09)	−0.42	0.695
**SW $Monofilaments_{Shoulder}$** *(pre-treatment)*	1.19 (0.18)	6.65	**0.003**
**R^2 (%)**	85.2%		
**Model**	F (4.17)= 12.46, **$p < 0.05$**		

Note: SW: Semmes-Weinstein; β: regression coefficient; SE: standard error.

Supplementary Table S5. Multivariable regression model of the FMA-UE$_{\text{Motor Function}}$ post-treatment score in the experimental group.

	**FMA-UE$_{\text{Motor Function}}$** (*post-treatment*)		
	**β (SE)**	***t***	***p value***
**Sex (Male vs Female)**	−7.25 (7.61)	−0.953	0.395
**Age (years)**	0.31 (0.30)	1.01	0.366
**Disease duration**	0.30 (0.85)	0.35	0.740
**FMA-UE$_{\text{Motor Function}}$** *(pre-treatment)*	0.95 (0.10)	8.87)	**0.001**
**R^2 (%)**	92.8%		
**Model**	F (4.17)= 26.87, **p< 0.005**		

Note: FMA-UE: Fugl Meyer Assessment Upper Extremity Scale; β: regression coefficient; SE: standard error.

Supplementary Table S6. Multivariable regression model of the MAL-14$_{\text{Amount scale}}$ post-treatment score in the experimental group.

	**MAL-14$_{\text{Amount scale}}$** (*post-treatment*)		
	**β (SE)**	***t***	***p value***
**Sex (Male vs Female)**	−22.49 (20.03)	−1.12	0.324
**Age (years)**	0.19 (0.80)	0.24	0.817
**Disease duration**	0.48 (2.17)	0.22	0.833
**MAL-14$_{\text{Amount scale}}$** *(pre-treatment)*	1.33 (0.50)	2.62	**0.044**
**R^2 (%)**	56.5%		
**Model**	F (4.17)= 2.604, p> 0.05		

Note: MAL-14: Motor Activity Log; β: regression coefficient; SE: standard error.

Supplementary Table S7. Multivariable regression model of the MAL-14$_{\text{How well scale}}$ post-treatment score in the experimental group.

	**MAL-14$_{\text{How well scale}}$** (*post-treatment*)		
	**β (SE)**	***t***	***p value***
**Sex (Male vs Female)**	−7.51 (20.62)	−0.36	0.734
**Age (years)**	−0.19 (0.85)	−0.23	0.828
**Disease duration**	−0.92 (2.30)	−0.40	0.708
**MAL-14$_{\text{How well scale}}$** *(pre-treatment)*	1.76 (0.69)	2.53	0.049
**R^2 (%)**	59.2%		
**Model**	F (4.17)= 2.28, p> 0.05		

Note: MAL-14: Motor Activity Log; β: regression coefficient; SE: standard error.

Supplementary Table S8. Multivariable regression model of the SIS-16 post-treatment score in the experimental group.

	**SIS-16** (*post-treatment*)		
	**β (SE)**	***t***	***p value***
**Sex (Male vs Female)**	8.66 (7.82)	1.10	0.330
**Age (years)**	−0.02 (0.30)	−0.07	0.944
**Disease duration**	−0.33 (1.02)	−0.32	0.761
**SIS-16** *(pre-treatment)*	0.90 (0.23)	3.77	**0.019**
**R^2 (%)**	75.7%		
**Model**	F (4.17)= 7.223 , **p< 0.005**		

Note: SIS-16: Stroke Impact Scale; Motor Activity Log; β: regression coefficient; SE: standard error.

Supplementary Table S9. Multivariable regression model of the MAL-14$_{\text{Amount scale}}$ post-treatment score between groups.

	**MAL-14$_{\text{Amount scale}}$** *(post-treatment)*		
	**β (SE)**	***t***	***p value***
**Sex (Male vs Female)**	−6.40 (6.42)	−0.99	0.337
**Age (years)**	0.03 (0.36)	0.08	0.934
**Disease duration**	0.09 (0.88)	0.10	0.918
**MAL-14$_{\text{Amount scale}}$** *(pre-treatment)*	1.19 (0.17)	6.99	**<0.001**
**R^2 (%)**	76.3%		
**Model**	F (4.17)= 14.83 , **p< 0.001**		

Note: SIS-16: Stroke Impact Scale; Motor Activity Log; β: regression coefficient; SE: standard error.

Supplementary Table S10. Multivariable regression model of the MAL-14$_{\text{How well scale}}$ post-treatment score between groups.

	**MAL-14$_{\text{How well scale}}$** *(post-treatment)*		
	**β (SE)**	***t***	***p value***
**Sex (Male vs Female)**	−6.14 (6.96)	−0.88	0.393
**Age (years)**	0.00 (0.39)	0.01	0.991
**Disease duration**	−0.06 (0.98)	−0.06	0.946
**MAL-14$_{\text{How well scale}}$** *(pre-treatment)*	1.23 (0.21)	5.80	**<0.001**
**R^2 (%)**	70.3%		
**Model**	F (4.17)= 11.50 , **p< 0.001**		

Note: SIS-16: Stroke Impact Scale; Motor Activity Log; β: regression coefficient; SE: standard error.
